# Ligaments of the Costovertebral Joints including Biomechanics, Innervations, and Clinical Applications: A Comprehensive Review with Application to Approaches to the Thoracic Spine

**DOI:** 10.7759/cureus.874

**Published:** 2016-11-11

**Authors:** Erfanul Saker, Rachel A Graham, Renee Nicholas, Anthony V D’Antoni, Marios Loukas, Rod J Oskouian, R. Shane Tubbs

**Affiliations:** 1 Department of Anatomical Sciences, St. George's University School of Medicine, Grenada, West Indies; 2 Department of Anatomy, The Sophie Davis School of Biomedical Education; 3 Department of Physical Therapy, Samford University; 4 Neurosurgery, Complex Spine, Swedish Neuroscience Institute; 5 Neurosurgery, Seattle Science Foundation

**Keywords:** costovertebral joint, spine, anatomy, thoracic

## Abstract

Few studies have examined the costovertebral joint and its ligaments in detail. Therefore, the following review was performed to better elucidate their anatomy, function and involvement in pathology. Standard search engines were used to find studies concerning the costovertebral joints and ligaments. These often-overlooked ligaments of the body serve important functions in maintaining appropriate alignment between the ribs and spine. With an increasing interest in minimally invasive approaches to the thoracic spine and an improved understanding of the function and innervation of these ligaments, surgeons and clinicians should have a good working knowledge of these structures.

## Introduction and background

The costovertebral joint ligaments are relatively unknown and frequently overlooked anatomical structures [1]. Although small and short in size, they are abundant, comprising 108 costovertebral ligaments in the normal human thoracic spine, and they are essential to its stability and function [2-3]. These ligaments are rarely at rest as they are frequently engaged in every respiratory effort that involves the movement of the thoracic cage [2]. As with every human structure, these ligaments are subject to pathology either from trauma or an intrinsic disease process. Additionally, with the increase in popularity of minimally invasive spine approaches, knowledge of these ligaments is important to spine surgeons. This article will provide an overview of the anatomy, physiology, biomechanics, innervation and the pathological conditions/states of these ligaments. Although much has been written and studied regarding the cervical and lumbar spinal ligaments, a relatively small amount of studies have focused on the thoracic costovertebral ligaments [4].

## Review

### Anatomy

**Figure 1:**
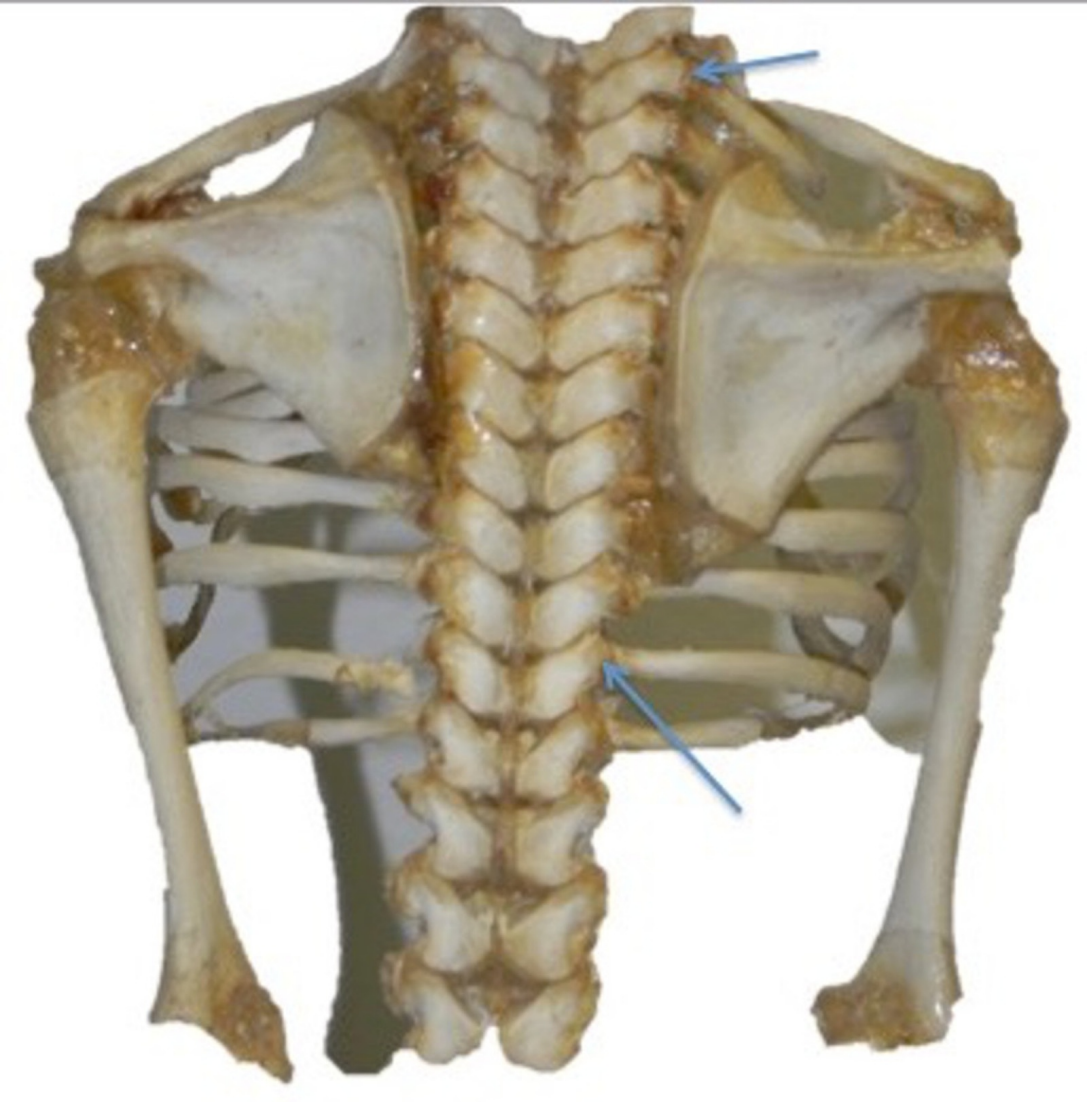
Fetal skeleton from a posterior view noting the lateral costotransverse ligaments (arrows).

**Figure 2:**
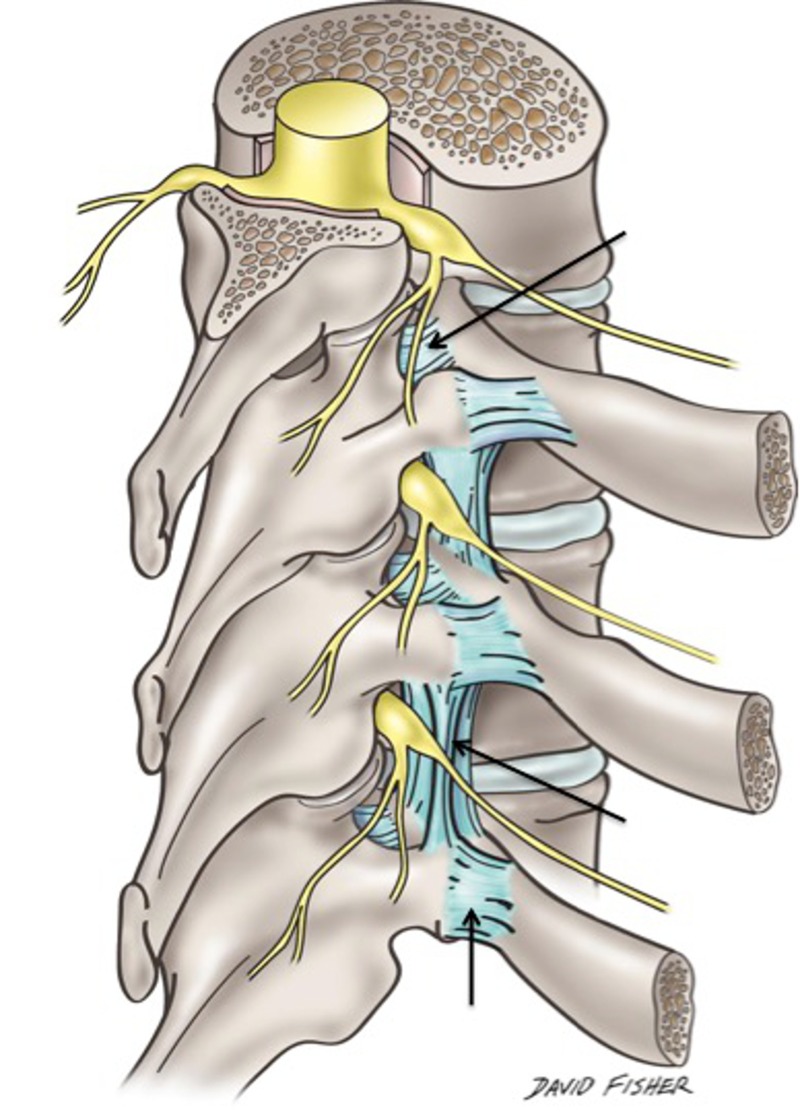
Schematic drawing noting various ligaments of the costovertebral joints: the costotransverse ligament (upper arrow), superior costotransverse (middle arrow) and lateral costotransverse ligament (lower arrow). Note the relationship of these ligaments to the dorsal root ganglia shown in this illustration.

**Figure 3:**
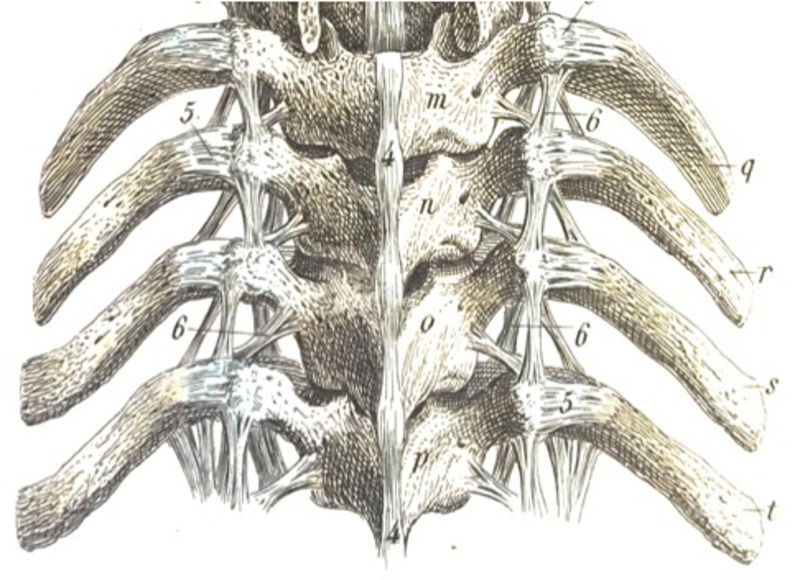
Posterior depiction of many of the larger and smaller costovertebral ligaments from Bock’s Hand-Atlas der Anatomie des Menschen (Verlag der Rengerschen Buchhandlung, Berlin, 1860).

**Figure 4:**
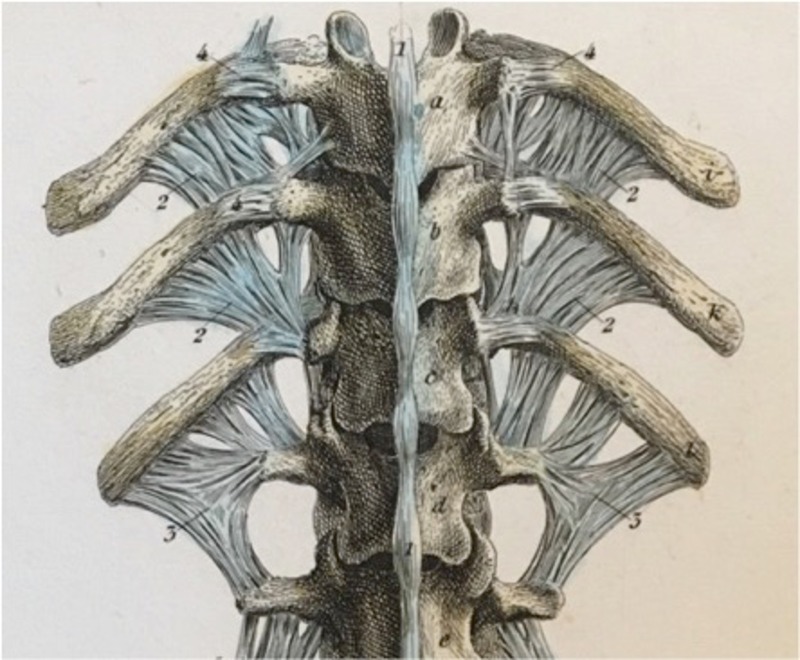
Alternative view of the posterior depictions from Figure 3.

**Figure 5:**
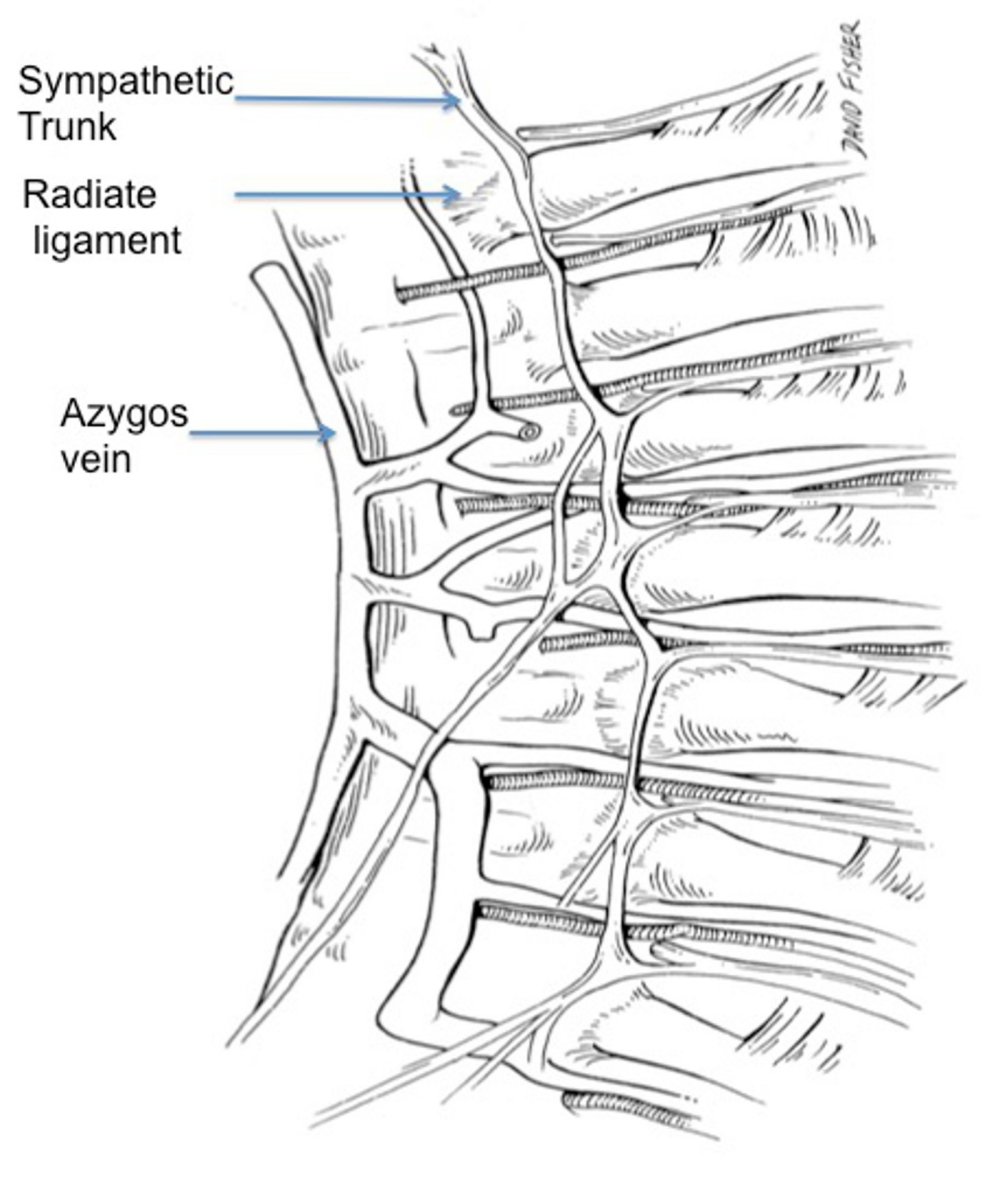
Schematic drawing of the internal aspect of the posterior thorax noting the radiate ligament attaching the head of the rib to the vertebral body. Note the relationship between this ligament and the overlying sympathetic trunk.

The costovertebral joints are arthrodial joints connecting the head of the ribs with the bodies of the thoracic vertebrae (Figures 1-4) [5]. The second to ninth ribs have articulating surfaces consisting of an inferior and superior costal facet connecting to the bodies of two adjacent vertebrae: the vertebra of the same number and the vertebra above. An intervertebral disc is positioned between these facets. A short horizontally placed intra-articular ligament extends from the ventral surface of the head of a rib to the intervertebral disc, which helps to delineate an upper from a lower joint cavity. This is covered entirely by an articular capsule, which becomes thickened anteriorly to form the radiate ligament (ligamentum capitis costae radiatum), attaching to the intervertebral disc and vertebral bodies (Figure 5) [6-7]. The heads of the first, 10th, 11th and 12th ribs each articulate with only one vertebra; thus, these joints only have a single cavity [6].

The articular surface of a rib tubercle articulates with the costal facet on the transverse process forming the costotransverse joint [6]. These joints have a small synovial cavity that is surrounded by a weak articular capsule, which is reinforced and strengthened by three accessory ligaments: the costotransverse (ligamentum costotransversarium), the lateral costotransverse (ligamentum costotransversarium laterale) and the superior costotransverse (ligamentum costotransversarium superius) [6-7]. These costotransverse ligaments connect the dorsal surface of the neck of the rib to the anterior surface of the transverse process of the vertebra. The lateral costotransverse ligaments connect the posterior surface of the tip of the transverse process to the nonarticular part of the tubercle of the analogous rib [5].

The superior costotransverse ligament connects the neck of a rib to the transverse process of the vertebra above. It is composed of two sets of fibers: anterior and posterior. The anterior set extends obliquely, superiorly and laterally from the crest on the superior border of the rib neck to the anterior surface of the transverse process of the vertebra immediately superior to it [5]. The posterior set extends superiorly and medially from the superior rib crest border to the inferior border of the transverse process of the vertebra immediately superior to it. The superior costotransverse ligament is frequently absent on the first rib [8]. Since the 11th and 12th ribs (“floating ribs”) do not have any anterior connection to the sternum, they are devoid of costotransverse joints [5]. The 12th rib is attached to the transverse process of the first two lumbar vertebrae by the lumbocostal ligament [7].

The costovertebral ligaments are dense, fibrous ligaments, and the majority of fibers run in a uniform direction. They have an ample amount of elastic fibers that stretch with movement in one direction and shorten with movement in the opposite direction, therefore remaining taut rather than becoming lax [7]. At the thoracic level, a direct ligamentous connection between the extraforaminal thoracic spinal nerves and surrounding structures exists, consisting of a superior and inferior component [9]. The superior costotransverse ligament encompasses the superior component, while the inferior component, called the inferior extraforaminal ligament, attaches the superior transverse process to the inferior transverse process. These ligaments are oriented perpendicularly to the thoracic spinal nerves. It is speculated that these ligaments add a protective mechanism against traction and compression of the nerves by maintaining proper positioning of the nerves in the intervertebral foramen [9].

### Physiology and biomechanics 

The skeletal makeup of the thorax includes the thoracic vertebrae, intervertebral discs, ribs, sternum and costal cartilages [6]. The contents of the hollow thorax consist of the lungs, heart and thymus gland, as well as the beginning of the great systemic artery (aorta) and the end of the great systemic veins (superior and inferior vena cava) [7]. The costovertebral joint and costovertebral ligaments play a pivotal role in thoracic stabilization, load bearing, mobility, protection and chest wall movement, all while contributing heavily to respiratory effort. The thoracic architecture allows motion in all planes. In flexion-extension, the range of motion begins at four degrees at T1 and increases incrementally to 12 degrees at T12. Lateral twisting typically allows six to seven degrees per vertebral segment. Rotation occurs inversely to flexion. T1 rotates nine degrees and incrementally decreases to two degrees at T12. Rib-joint stiffness is greatest at T2 and weakest at T10 [10].

The thoracic cage plays an important role in load-bearing, providing between 30-40% of thoracic spine stiffness [11]. Additionally, the costovertebral complex serves as scaffolding for the musculature of the thoracic spine and shoulder. All of the above motions and loads cause stress to the costovertebral attachments at both the vertebral body and the transverse process. Fortunately, the strong costovertebral and costotransverse ligaments stabilize these joints and also add a degree of flexibility [10].

The costovertebral complex is mandatory for respiratory movement of the chest wall. There are two basic mechanisms for lung expansion and contraction; one is the caudal and cephalic movement of the diaphragm, which lengthens or shortens the chest cavity. The contraction of the diaphragm during inspiration generates a negative intrathoracic pressure, allowing for expansion of the lungs. During quiet breathing, the intrapleural pressure is about -2.5 mm Hg (relative to atmospheric pressure) at the start of inspiration and decreases to approximately -6 mm Hg. Thus, generating a slightly negative pressure gradient allows air to flow in. When the diaphragm relaxes, the elastic recoil of the lungs, abdominal structures and chest wall compresses the lungs, causing expiration. The airway pressure becomes slightly positive at that point, allowing air to flow out of the lungs. During quiet expiration, contraction of the inspiratory muscles in the early stages exerts a braking action on the recoil forces and delays expiration [12]. Quiet breathing occurs primarily by diaphragmatic movement. Even bilateral paralysis of the diaphragm does not lead to hypoventilation as long as the thorax remains mobile and the thoracic muscles and costovertebral joints are functioning well. However, the diaphragm is instrumental in the event the intercostal muscles are paralyzed or the thorax becomes immobile [13].

The second method for lung expansion is the elevation of the ribs, which increases the anteroposterior and transverse diameter of the chest cavity. In the normal resting position, ribs are slanted downward allowing the sternum to be situated posteriorly. When the ribs are elevated, they are projected laterally and anteriorly leading to an increased transverse and anteroposterior diameter. With an increased diameter, maximum inspiration can increase the intrathoracic volume by as much as 20% [13].

The muscles of deep inspiration involved in raising the rib cage are the external intercostals, anterior serrati, scalene, sternocleidomastoid, levatores costarum and the serratus posterior superior [5]. As these muscles contract, the ribs and sternum are elevated and projected outward. In a forced inspiration, the muscles of deep inspiration are utilized concurrently with the levator scapulae, trapezius and rhomboids to raise and fix the scapula. Additionally, the pectoral muscles and serratus anterior muscles also aid in elevating the ribs [5]. Strong inspiration can reduce intrapleural pressure as low as -30 mm Hg [12].

The muscles of forced expiration that pull the rib cage downward are the internal intercostals and the abdominal recti primarily, with minor contributions from the quadratus lumborum, subcostals, transverse thoracic and serratus posterior inferior [5]. The posterior costovertebral joints and the anterior costochondral joints assist in making the downward movement possible. The range of motion at any one of the thoracic joints is small, but the frequency of movement of these joints is extremely great [6]. The second through the sixth ribs each move around two axes. Movement at the costovertebral joint in a side-to-side (gliding) axis results in the raising and lowering of the sternal end of a rib. This is referred to as the “pump-handle” movement. Because the ribs are sloped downward, any elevation (e.g., during deep inspiration) will result in an upward (cephalic) and forward (anterior) movement of the sternum, thus increasing the anteroposterior diameter of the thorax [6]. The lower ribs move laterally when they are elevated, which consequently increases the transverse diameter of the thorax. This motion primarily occurs at the seventh through 10th costotransverse joints. Since the articular tubercle of the joints is flat, the ribs move up and down and allow what is referred to as the “bucket-handle” movement.

The axis of rotation at the first rib is unique compared to the other ribs. There is minimal movement at the first rib during quiet breathing. A slight rotation about its neck causes a small amount of rising and lowering of the sternum, which can result in a minute change of the anteroposterior diameter of the thoracic inlet [6]. The second through sixth ribs move in a similar axis to the seventh through 10th ribs. The costal cartilage deviates upwards on the second through sixth ribs, which causes a posterior movement of the sternum (the “pump handle” effect) during elevation [6]. The length of the transverse process also has an effect on the costovertebral complex. As the length of the transverse process varies (with its concomitant costotransverse articular surface), the leverage that is exerted on the costovertebral joints varies as well [2]. Movement a few millimeters anteriorly, cephalically or laterally of the thoracic wall can increase its volume by approximately one-half liter [6].

The costovertebral complex is an essential component of the biomechanics of chest wall movement. The costovertebral ligaments make the actions of the costovertebral joints and intervertebral movement possible. These ligaments function to affix, stabilize and allow some motion of the ribs on the thoracic vertebra at the costovertebral joint. Their presence helps with the load-bearing, protection, posture and scaffolding roles that the thoracic cage provides with their stabilization properties [14]. More so, they allow and limit movement of the ribs at the transverse joint to allow for maximum expansion of the thoracic cavity as needed for respiratory demand [11]. Lastly, their actions on both the costovertebral and intervertebral complexes allow lateral bending and axial rotation.

### Innervation

Innervation of the costovertebral ligaments is supplied by the lateral branch of the thoracic dorsal rami of C8 and T1 to T11 [15]. The costovertebral joints receive this innervation in a segmental fashion with each joint receiving fibers from the level above and directly below it. Pain originating from the joints is well-localized and level specific [10]. This seems to follow Hilton’s law, which states “that the innervation of a joint is the same innervation as the muscles which move the joint and the skin overlying the joint” [15].

Mechanoreceptors have been identified in the region of the middle costotransverse ligament [16]. This innervation is characteristic of neurons providing nociception and mechanoreceptor activity. Activation of these mechanoreceptors, either by mechanic loading or inflammation, may cause pain and reflex muscular hypertonus. Tachykinins (substance P and calcitonin gene-related peptide), neuropeptides activated in response to nociceptive stimulation, have been found aside costovertebral ligaments. Thus, it appears that known neuropeptides are present along the costovertebral ligaments that can activate pain and propagate an inflammatory cascade [16].

Studies in rabbits have identified slowly adapting mechanoreceptors in the costovertebral complex that are capable of signaling the absolute rib position, direction and velocity of movement [17]. These mechanoreceptors are also sensitive to tension and can alter the strain of the surrounding ligaments. There is a group of these receptors that is sensitive to caudal rib movement, known as “expiratory receptors,” and a group that is sensitive to rostral rib movement, or “inspiratory receptors” [17]. There have been no similar studies in humans. Nonetheless, these findings provide a probable pathway to consider. In approximately 60% of individuals, there is a linkage of the brachial plexus to the first and/or second intercostal nerve, known as Kuntz’s nerve. Therefore, disorders affecting the first or second costovertebral joints can result in arm pain referred via this pathway [10].

### Clinical implication

Determining the etiology of posterior thoracic pain can be difficult [1]. The neural complexity of the thoracic spine, along with referred visceral pain makes the differential diagnosis difficult [15]. The differential diagnosis for posterior thoracic pain is broad and includes vertebral/rib fracture, intervertebral disc protrusion/herniation, spinal stenosis, spinal nerve irritation/entrapment, paraspinal muscle or ligament strain, diffuse idiopathic skeletal hyperostosis, intercostal neuralgia, upper thoracic syndrome (T4 syndrome), ankylosing spondylitis, osteoarthritis, zygapophyseal joint dysfunction, active trigger points, visceral referred pain of cardiac, aortic, renal, pulmonary, esophageal, gallbladder or hepatobiliary origin and metastatic malignant lesions, among others [1].

Properly diagnosing the causative origin of posterior thoracic pain can involve costly tests, substantial time investment and in some cases, can avert a disastrous outcome [16]. Of the list above, the conditions directly involving the costovertebral ligaments are costovertebral/costotransverse joint dysfunction, rib neck fractures, osteoarthritis, ankylosing spondylitis and other spondyloarthritis. However, there are other inflammatory and traumatic states that can affect the costovertebral ligaments. The most common state is straining of the costovertebral/costotransverse joint complexes as a result of hyperextension of the ligament [14]. This type of injury occurs after a sudden movement involving twisting, bending or overextension of the spine [1]. Benign events such as sneezing, coughing or postural strain can also activate nociceptive receptors and result in pain, muscle spasm and disability [10].

Costovertebral strains generally occur when compression and torsional forces applied to the joint are greater than the joint can withstand, inflicting damage to the ligaments, cartilage or both. In extreme cases, the head of the rib may dislocate [1]. The majority of posterior rib fractures occur at the rib neck between the costovertebral and costotransverse joint [10]. This is probably due to the strong costovertebral ligamentous attachment to the rib head and tubercle, with its propensity to injury owing to its presence on either side of the rib neck. Pain with costovertebral joint strain is usually unilateral, occurring with movement of the spine, especially twisting and lateral bending. Deep breaths, coughing and sneezing may elicit pain, which may radiate to the scapular region or anterior chest wall [1].

Osteoarthritis is a degenerative joint disease resulting from the breakdown of articular cartilage and underlying erosion of subchondral bone and synovium. It is believed to be caused by mechanical stress on the joint and low-grade inflammatory process [18]. Osteoarthritis of the costovertebral joints is quite common. One extensive cadaver study found a 48% prevalence of osteoarthritis of the costovertebral joints. Pain due to osteoarthritis is typically deep, achy joint pain exacerbated by extensive use, reduced range of motion and stiffness during rest [1]. Its spectrum can vary from asymptomatic to severely debilitating [19].

Spondyloarthritis is a family of chronic inflammatory diseases that involves both the joints and the entheses; it includes ankylosing spondylitis, psoriatic arthritis, reactive arthritis, arthritis associated with inflammatory bowel disease and undifferentiated spondyloarthritis [20]. Ankylosing spondylitis primarily affects the axial skeleton (e.g., sacroiliac joints and spine) [21]. The disease prevalence is one to three per 1000 in the general population with a typical age of onset between the second and fifth decade, affecting more men than women in a 2:1 ratio [22-23]. Ankylosing spondylitis alters the biomechanical properties of the spine via chronic inflammatory process (of unknown etiology), which yields a brittle, minimally compliant spinal column [24]. Involvement of the costovertebral and costotransverse joints results in the rigidity of the chest wall and subsequent inability to fully expand the chest on inspiration. This leads to a significant reduction in exercise capacity [21]. Furthermore, in ankylosing spondylitis, there is ossification of the spinal ligaments (including the costovertebral and costotransverse), resulting in a fixed kyphotic thoracic spine. Lastly, an ankylosed noncompliant rib cage may lead to a restrictive lung disease pattern [24]. These are a few of the conditions and diseases that can affect the costovertebral and costotransverse ligaments.

**Figure 6:**
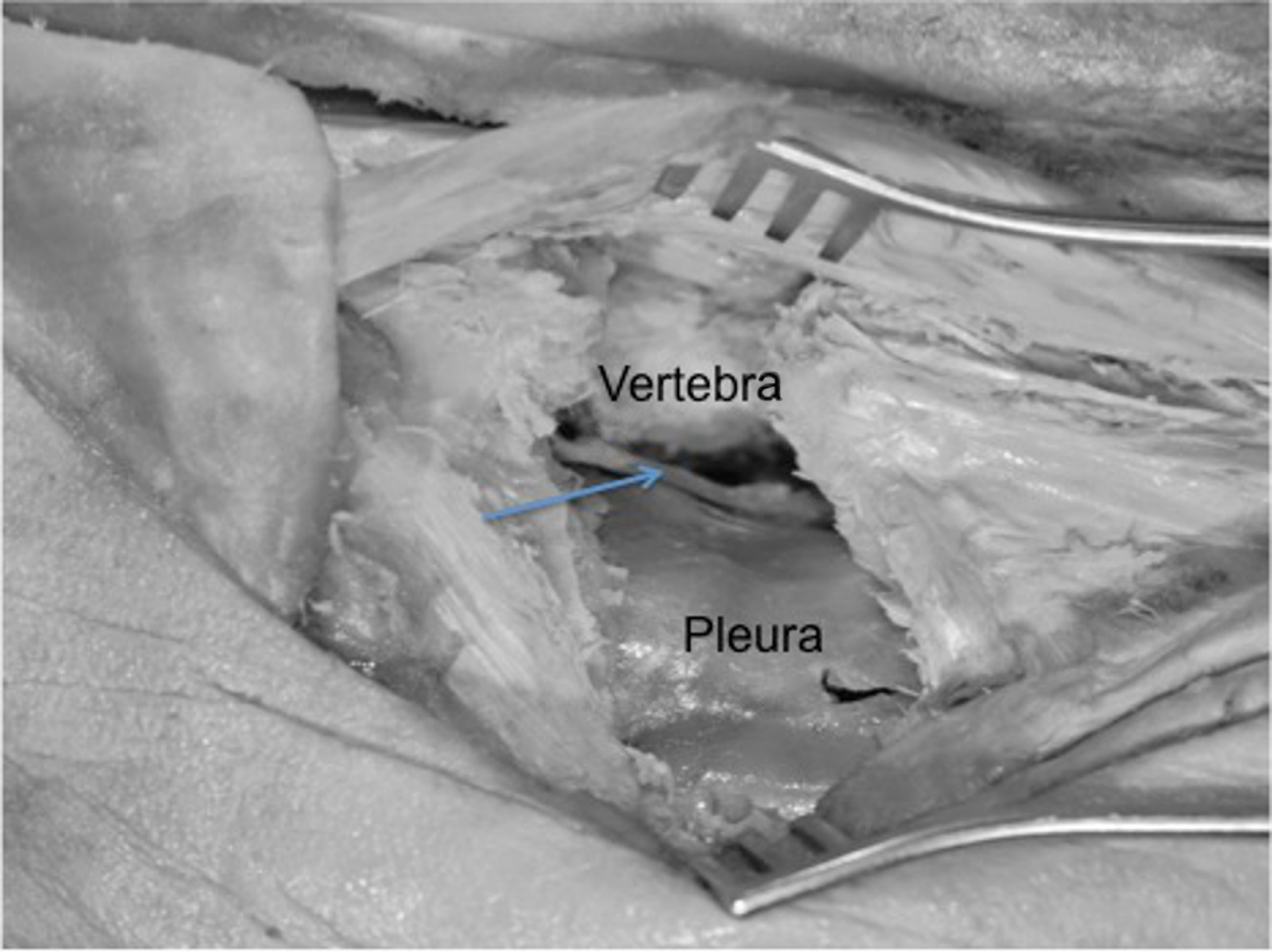
Left posterior costotransversectomy procedure where the proximal rib and transverse process and ligaments have been removed to access the anterolateral vertebral body (vertebra) and posterior thorax. Note the intact pleura and exposed sympathetic trunk (arrow).

Approaches to the thoracic spine, especially lateral approaches (e.g., thoracic osteotomies), might encounter several of the ligaments connecting the spine to the ribs (Figure 6). Knowledge of these and their relationships to the nearby neural elements is important to a spine surgeon. Procedures in this region include costotransversectomy for lateral access to the spinal canal.

## Conclusions

An understanding of the anatomy, physiology, biomechanics and innervation of the costovertebral joint ligaments is necessary to properly evaluate, differentiate, exclude, diagnose and render appropriate treatment for a variety of pathophysiological states involving the posterior thorax.
